# Effects of Carbon Dioxide on Hemolymph and Brain Proteomes in Honey Bee Workers (*Apis mellifera* L.)

**DOI:** 10.3390/insects17060630

**Published:** 2026-06-15

**Authors:** Ying Wang, Beibei Ma, Yu Fang

**Affiliations:** 1 Key Laboratory of Agri-Products Quality and Biosafety, Anhui Agricultural University, Ministry of Education, Hefei 230036, China; 2 State Key Laboratory of Resource Insects, Institute of Apicultural Research, Chinese Academy of Agricultural Sciences, Beijing 100193, China

**Keywords:** carbon dioxide, honey bee, hemolymph, brain, proteomes

## Abstract

Carbon dioxide (CO_2_) is essential in beekeeping practices but its optimal dosage and physiological effects on honey bees remain unclear. This study examined CO_2_ tolerance and molecular responses across three developmental stages: newly emerged, nurse, and forager bees, using gradient exposure and proteomic analysis. Newly emerged bees demonstrated the highest CO_2_ tolerance. Hemolymph and brain exhibited distinct responses: the cytochrome P450 pathway dominated in hemolymph, while the brain displayed stage-specific strategies. Newly emerged bees activated metabolic reorganization and clearance pathways. Nurse bees strengthened antioxidant defenses, while foragers enhanced amino acid metabolism to produce antioxidant precursors. All stages showed role-specific energy metabolism reprogramming to meet increased post-exposure demands. These findings provide critical data and theoretical foundations for honey bee colony management, transportation, and handling practices. The results also contribute valuable insights to the fundamental biology of other insects.

## Simple Summary

Carbon dioxide is widely used in beekeeping and research, yet its precise effects on honey bees at different life stages remain unclear. This study investigated how honey bees of different ages—newly emerged bees, nurse bees, and foragers—tolerate and respond to carbon dioxide exposure. We discovered that honey bees employ distinct, age-dependent strategies to cope with this stress. Newly emerged bees are the most resilient, protecting themselves through specialized chemical pathways in their blood and brain. Nurse bees show lower blood responses but prioritize brain defense, while foragers rely on blood detoxification and brain energy regulation. Across all stages, bees strategically redirect their energy resources to survive the stress. These findings provide a crucial understanding of how insects adapt to environmental stress. Practically, this research offers valuable data to improve honey bee colony management, safe transportation, and overall beekeeping practices, ultimately supporting the health of these essential pollinators.

## 1. Introduction

Honey bee is one of the most efficient pollinators in the world. Approximately 90% of wild plants and more than one-third of crops worldwide depend on animal-mediated pollination to enhance yield and quality [1,2]. Among these, around three-quarters of the crops rely on bee pollination for increased production. It is estimated that bee pollination contributes nearly 153 billion euros to the global economy [3,4]. Among the pollinating bees, European honey bees (*Apis mellifera* L.) are highly popular with beekeepers due to their gentle nature and high production. Furthermore, European honeybees produce a wealth of products such as honey, pollen, royal jelly, and beeswax, which benefit health of human, as well as the economic benefits brought by pollination.

With recent industrialization and urbanization, greenhouse gas emissions—particularly carbon dioxide (CO_2_), which accounts for a significant portion of these gases—have been steadily increasing [5]. The resulting climate change has altered the habitats of many species, including honey bees [6]. To monitor subtle CO_2_ fluctuations in both external and in-hive environments, bees have evolved specialized CO_2_ receptors on their antennae, located within the olfactory receptor neurons [7,8]. CO_2_ serves as a critical sensory cue for their activities [9]; for instance, CO_2_ exposure can alter the foraging preferences of worker bees for pollen and nectar [10].

Moreover, CO_2_ significantly influences both the short-term and long-term memory of worker bees, thereby affecting their learning and foraging behaviors. Specifically, elevated CO_2_ concentrations have a clear negative impact on their memory capacity [11]. Indirectly, however, atmospheric CO_2_ affects the nectar-producing plants that provide food for bees [12]. Under environmental heat stress, increased CO_2_ levels can accelerate sucrose and starch metabolism, promoting pollen germination and production in various wild plants. This, in turn, may eventually benefit foraging efficiency and colony reproduction [13,14].

Beyond these external effects, honey bee behaviors are closely tied to CO_2_ concentrations within the colony. The CO_2_ level in the hive rises when a colony is threatened; for instance, exposure to *Varroa destructor* mites and pesticides increases oxygen consumption and CO_2_ emissions, leading to higher internal concentrations than those found in healthy hives [15,16,17]. Remarkably, bees exhibit self-defense behaviors against parasitic invasions by actively manipulating the hive’s CO_2_ levels. Specifically, bees can restrict ventilation to promote CO_2_ accumulation as a means of mite control [16]. At approximately 3.8% CO_2_, mite mortality increases to 37 ± 4.2%, compared to 23 ± 4.2% under standard ventilation, providing a selective advantage against parasites without harming the bees themselves [15].

Furthermore, honey bee colonies actively regulate internal CO_2_ concentrations to maintain a stable living environment. When CO_2_ levels rise due to collective respiratory metabolism and reach a specific threshold, it triggers fanning behavior in worker bees [18,19]. Positioned near the hive entrance, these bees vibrate their wings to facilitate air circulation between the internal and external environments, effectively removing excess. Through this ventilation, the colony maintains CO_2_ levels between 0.10% and 4.25% [20]. This concentration is also closely correlated with hive temperature and humidity. For instance, when the hive temperature remains below 11 °C, CO_2_ is released continuously; however, once temperatures exceed 11 °C, bees transition to intermittent CO_2_ emission [21,22]. Conversely, if the growth and development of the colony are severely disrupted, a characteristic decrease in hive CO_2_ concentration can be observed [23].

For scientific purposes, CO_2_ is frequently utilized as an anesthetic for bees due to its safety, convenience, and flexibility. Primarily, CO_2_ facilitates oviposition in queens; repeated CO_2_ exposure has been shown to exert a significant effect on the initiation of egg-laying in *Apis mellifera* L. [24,25]. Similar stimulatory effects were observed in *Apis cerana* queens, characterized by higher vitellogenin gene expression following CO_2_ treatment [10,26]. However, CO_2_ exposure also has a series of negative physiological impacts on honey bees. Frequent CO_2_ treatment is associated with a significantly shortened lifespan [27,28]. Furthermore, CO_2_ anesthesia can reduce foraging behavior, decrease the body weight and water content of adult bees, and accelerate the aging process [28,29].

CO_2_ also impacts other bee species. In hornets, specific concentrations of CO_2_ exhibit a lethal effect when combined with high temperatures [30]. Conversely, for bumblebee queens, CO_2_ treatment—when paired with low temperatures—can promote egg-laying [31,32]. At the molecular level, CO_2_ enhances fat body function by accelerating metabolism, which leads to decreased lipid content in the fat bodies and increased glycogen and protein levels in the ovaries [33]. Furthermore, CO_2_ stimulates the expression of genes related to foraging and flight, such as HR38 (hormone receptor 38) and Egr1 (early growth response gene 1), within the brain neural cells of *Bombus ignitus* L. [34].

Due to its close association with bee colony activity, CO_2_ is widely utilized in both laboratory and apiary settings. CO_2_ is frequently employed as an anesthetic agent. Its primary applications include immobilizing queens during instrumental insemination, temporarily calming aggressive colonies during extensive hive inspections or long-distance transportation, and facilitating the safe collection of worker bees for venom extraction or experimental sampling. However, the optimal exposure duration and the corresponding molecular impacts of CO_2_ on honeybees remain to be fully elucidated. Given that the complex division of labor in honeybees may lead to differential responses to CO_2_ exposure, this study selected representative worker bees—specifically emerging bees, nurse bees, and forager bees—as experimental subjects. Hemolymph serves as a critical indicator of a honeybee’s physiological state [35], playing a vital role in nutrient transport, ensuring adequate energy supply to organs, and regulating physiological development and innate immune defense [36]. Concurrently, the brain, as the nerve center, directly mediates learning and memory functions, ultimately governing behavior [37]. Therefore, to further investigate the molecular influence of CO_2_ exposure, we collected hemolymph and brain samples from these bees for proteomic analysis. This research aims to determine the practical tolerance threshold of worker bees to CO_2_ treatment and to decode the molecular mechanisms underlying their response to external environmental stimuli. The findings are expected to enhance our understanding of honeybee adaptation biology and provide practical, directive significance for the preservation and transportation of bee colonies.

## 2. Material and Methods

### 2.1. Chemical Reagents

All chemicals were purchased from Sigma-Aldrich (St. Louis, MO, USA) unless otherwise stated.

### 2.2. Honey Bee Collection and Life Stage Categorization

Honey bee (*Apis mellifera* L.) colonies with identical strengths were raised in the apiary of Institute of Apicultural Research, Beijing. The internal structure of the colony is stable and exhibits healthy development. Experimental samples were collected according to the following criteria: newly emerged bees (E) refer to bees that have just emerged from their cells; nurse bees (N) are those capable of secreting royal jelly; and forager bees (F) are identified as bees returning to the hive with pollen loads on their hind legs. Each sample was collected from 3 colonies.

### 2.3. CO_2_ Exposure and Resuscitation State Determination

**Figure 1:**
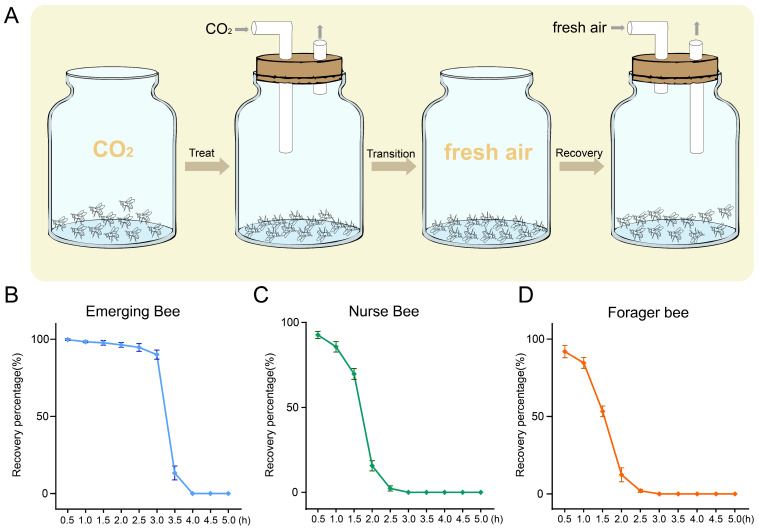
Carbon dioxide (CO_2_) exposure and recovery protocol for honey bee workers. (**A**) Schematic of the experimental design. Groups of 100 bees were placed in a 1000 mL jar and exposed to a constant flow of CO_2_ (35 mL/min) at 34 °C for a designated duration. Following exposure, bees recovered for 3 h in a container with continuous fresh air, with their status recorded hourly. Control groups were handled identically but without CO_2_ exposure. (**B**–**D**) Recovery rate curves for newly emerged (**B**), nurse (**C**), and forager (**D**) bees during the 3 h post-exposure recovery period.

To assess the critical CO_2_ tolerance threshold in different worker castes, newly emerged (E), nurse (N), and forager (F) bees were subjected to CO_2_ treatment for varying durations, with time points set every 30 min for up to 5 h. For each replicate, 100 bees were placed in a 1000 mL jar filled with 99.9% CO_2_. The jar was then sealed and maintained with a constant flow of CO_2_ (35 mL/min) inside an incubator at 34 °C for the designated exposure time (Figure 1A). High-purity CO_2_ (99.9%) was supplied from compressed gas cylinders (Beijing Qianxi Jingcheng Gas Co., Ltd., Beijing, China). To maintain a constant flow of CO_2_ at 35 mL/min, a precision mass flow controller (ACU20FD-L, Beijing Accuflow Technology Co., Ltd., Beijing, China) was employed to regulate the gas flow rate. One end of the flow controller was connected to the CO_2_ gas cylinder, while the other end was connected via sterile silicone tubing to the experimental jars placed inside the incubator (HERACELL 150i, Thermo Fisher Scientific, Waltham, MA, USA). This setup ensured stable and continuous delivery of CO_2_ gas into the jars maintained at 34 °C throughout the experimental period. Following exposure, bees were transferred to a recovery container with a continuous fresh air supply for 3 h, and their status was recorded hourly. Control groups were handled identically but without CO_2_ exposure.

To evaluate the effects of CO_2_ treatment, bees were divided into “recovered” and “non-recovered” groups post-exposure. The recovered group exhibited normal behaviors such as leg mobility, antennal movement, and abdominal respiration, while the others were classified as non-recovered. For practical applications in beekeeping, a critical threshold was defined as the time point where 90% of the bees successfully recovered. The bee recovery rate was then calculated and presented as the mean ± standard deviation from three independent replicates (n = 3) for each time point. Data analysis and visualization were performed using GraphPad Prism 9.0 (GraphPad Software, San Diego, CA, USA).

### 2.4. Tissue Dissection

#### 2.4.1. Hemolymph Sampling

Hemolymph was collected immediately following the collection of bee samples. First, an intact antenna was carefully detached from the head of the bee. Gentle pressure was then applied to the thorax, causing hemolymph to exude from the antennal base [38]. The droplets were immediately collected with a pipette, transferred to a 200 µL tube, and categorized by experimental group. All samples were subsequently stored at −80 °C for further analysis.

#### 2.4.2. Brain Sampling

To obtain brain samples, individual honeybees were first immobilized on a wax plate with a dissecting needle. The head capsule was then opened using fine forceps to fully expose the brain. After carefully detaching it from surrounding tissues, the compound eyes, ocelli, and adjacent glands were removed. The isolated brain tissue was collected and immediately frozen at −80 °C for subsequent analysis.

### 2.5. Protein Extraction

Proteins were extracted from the collected hemolymph and brain tissues. Samples were first thawed on ice, and a lysis buffer was added. The brain tissue was ground into fragments and both sample types were fully homogenized. The resulting lysate was sonicated for 10 min at 4 °C. Following sonication, the extract was clarified by centrifugation at 12,000 rpm for 15 min at 4 °C. The supernatant, containing the soluble protein fraction, was carefully transferred to a new tube.

To precipitate the proteins, four volumes of pre-chilled acetone were added, and the mixture was incubated for 20 min. The mixture was then centrifuged, and the acetone supernatant was discarded. The resulting protein pellet was resolubilized in a urea-based solution. A final centrifugation step was performed at 12,000× *g* for 15 min at 4 °C to remove any remaining insoluble debris. The supernatant containing the purified proteins was collected, and the protein concentration was quantified using the Bradford assay [39].

### 2.6. Peptide Preparation

For enzymatic digestion, a 50 µg aliquot of each protein sample was processed. Proteins were first reduced with 100 mM dithiothreitol (DTT) for 60 min at room temperature. Subsequently, cysteine residues were alkylated by adding 100 mM iodoacetamide (IAA) and incubating for one hour in darkness.

Prior to digestion, the sample was diluted with 50 mM ammonium bicarbonate (containing 10 mM glycine) to reduce the urea concentration to below 0.5 M. Trypsin was then added at a 50:1 protein-to-enzyme mass ratio, and the digestion was performed overnight (12–14 h) at 37 °C. The reaction was quenched by the addition of 1 µL of formic acid (FA).

The resulting peptides were desalted using C18 solid-phase extraction cartridges. Purified peptides were dried to completion in a vacuum concentrator (RVC 2-18, Martin Christ, Osterode am Harz, Germany) and subsequently reconstituted in 0.1% FA. The final peptide concentration was measured spectrophotometrically (NanoDrop 2000, Thermo Fisher Scientific, Waltham, MA, USA) and adjusted to 0.5 µg/µL.

### 2.7. LC-MS/MS Analysis

Peptide analysis was performed on an Orbitrap Exploris 240 mass spectrometer coupled to an Easy-nLC 1200 system (Thermo Fisher Scientific, Waltham, MA, USA). For each run, a 4 µL sample was loaded onto an Acclaim PepMap100 trap column (2 cm × 100 µm, C18, 3 µm; Omics Technology Co., Ltd., Beijing, China) at a flow rate of 500 nL/min.

Peptides were subsequently separated on an EASY-Spray analytical column (15 cm × 150 µm, C18, 2 µm; Omics Technology Co., Ltd., Beijing, China) using a 60 min gradient. The mobile phases consisted of 0.1% formic acid (FA) in water (Solvent A) and 0.1% FA in 80% acetonitrile (ACN) (Solvent B). The gradient profile was as follows: 8% B for 2 min; 8–12% B over 38 min; 12–30% B over 11 min; 30–45% B over 3 min; and a final ramp to 90% B over 6 min.

The mass spectrometer was operated in data-dependent acquisition (DDA) mode. Full MS scans were acquired in the Orbitrap from *m*/*z* 350 to 1500 at a resolution of 120,000, with an automatic gain control (AGC) target of 2.0 × 10^6^ and a maximum injection time (IT) of 50 ms. Precursor ions were fragmented via higher-energy collisional dissociation (HCD). The resulting dd-MS^2^ spectra were acquired at a resolution of 15,000, with an AGC target of 7.5 × 10^4^ and a normalized collision energy (NCE) of 30%.

### 2.8. Data Analysis

Raw data were processed with PEAKS Studio 8.5, searching against the *Apis mellifera* (NCBI, April 2024) and cRAP (April 2022) protein databases. Key search parameters were: 10 ppm precursor and 0.02 Da fragment ion mass tolerance, tryptic digestion with ≤2 missed cleavages, fixed carbamidomethylation of cysteine (+57.02 Da), and variable oxidation of methionine (+15.99 Da). Identifications required ≥2 unique peptides with the false discovery rate (FDR) < 1% at both peptide and protein levels.

For label-free quantification, a mass error tolerance of 10 ppm and a retention time shift tolerance of 6.0 min were applied. Only unique peptides were employed for protein quantification, with protein abundance derived from the summation of extracted ion chromatogram peak areas of the corresponding peptides. Data from three biological replicates were normalized to correct for inter-run variability, and protein expression levels were defined as the median of the resulting peptide ratios. The PEAKS Q quantification module was used to assess statistical significance through Benjamini–Hochberg FDR correction, employing cutoff values of FDR < 0.01 and fold change ≥ 1.5. The proteomics data have been deposited to the ProteomeXchange Consortium via the iProX repository under the identifier PXD068199 (http://proteomecentral.proteomexchange.org, accessed on 8 September 2025) [40,41].

To identify statistically significant biological pathways, pathway enrichment analysis was performed on the set of identified proteins using the KEGG Orthology-Based Annotation System (KOBAS 3.0; http://bioinfo.org/kobas, accessed on 8 September 2025) [42,43]. Protein accessions were mapped against the KEGG database, and enrichment was assessed using a hypergeometric test. The Benjamini–Hochberg procedure was employed to adjust *p*-values and calculate the False Discovery Rate (FDR, q-value). Significant pathway enrichment was defined as q-value < 0.05.

To explore functional relationships among the proteins, a protein–protein interaction (PPI) network was constructed using the GeneMANIA application in Cytoscape (version 3.9.1). The network was built using both known and predicted interaction data for *Drosophila melanogaster* (database version as of May 2025).

### 2.9. Western Blotting

To validate the quantitative proteomics data, Western blotting was performed according to established protocols [43]. For each sample, 20 µg of total protein was loaded in triplicate and separated by SDS-PAGE on a 4% stacking gel and a 12% resolving gel.

Following electrophoresis, proteins were transferred to a polyvinylidene difluoride (PVDF) membrane using an iBlot Gel Transfer System (20 V, 7 min; Invitrogen, CA, USA). The membranes were then blocked for one hour at room temperature with 5% non-fat milk in Tris-buffered saline with Tween 20.

Membranes were incubated overnight at 4 °C with a primary antibody against alcohol dehydrogenase class-3 (1:5000 dilution; Abcam, Cambridge, MA, USA). For normalization, a primary antibody against β-actin (1:5000 dilution; Abcam) was used as a loading control. After washing, membranes were incubated with the appropriate horseradish peroxidase (HRP)-conjugated secondary antibody (1:5000 dilution).

Protein bands were visualized using a chemiluminescence substrate, and images were captured with an E-BLOT imaging system (e-BLOT Life Science, Shanghai, China). Densitometric analysis was performed using ImageJ software (version 1.53k, National Institutes of Health, Bethesda, MD, USA) to quantify band intensities.

## 3. Results and Discussion

Our investigation began by evaluating the CO_2_ tolerance of newly emerged, nurse, and forager bees. A key finding was that newly emerged bees are remarkably tolerant, with 90% surviving up to three hours of CO_2_ exposure. This tolerance was significantly higher than that of nurse and forager bees, suggesting a robust, age-specific physiological defense mechanism in the youngest adults. This heightened resilience may be linked to the profound metabolic and cellular reorganization that occurs shortly after emergence, a hypothesis we subsequently explored using proteomics. A total of 6078 proteins (2002 protein groups) was identified, including 1831 proteins (950 protein groups) in the hemolymph and 5760 proteins (2147 protein groups) in the brain. The proteomic results revealed the molecular strategies hemolymph and brain adopted to deal with CO_2_ stress in different stages of workers.

### 3.1. Emerging Bees Exhibit Higher Tolerance to CO_2_ Exposure Compared to Nursing Bees and Forager Bees

Consistent with our expectations, honey bees with different labor divisions responded differently to CO_2_ exposure. Compared to nurse and forager bees, newly emerged bees exhibited the highest tolerance, sustaining CO_2_ exposure for up to 3 h with a 90% recovery rate (Figure 1B). In contrast, both nurse and forager bees had a much shorter tolerance time of 0.5 h (90% recovery rate, Figure 1C,D). Subsequent biological pathway analysis indicated that this high tolerance is likely related to a lower metabolic rate [44]. When compared to the control group, the CO_2_-treated group of newly emerged bees showed down-regulated expression of genes involved in respiratory metabolism pathways, such as the tricarboxylic acid (TCA) cycle, pentose and glucuronate interconversions, and pyruvate metabolism. Within the TCA cycle, the expression of succinate-CoA ligase [ADP/GDP-forming] subunit alpha, mitochondrial—a key regulatory protein—was lower in the treated group of emerged bees. As a key catalyst in the TCA cycle, this enzyme’s reaction generates high-energy phosphate compounds crucial for cellular energy metabolism [45,46]. Therefore, the impact of CO_2_-induced hypoxic stress on younger bees is likely attenuated by this metabolic suppression.

### 3.2. Cytochrome P450 Pathway Contributes to the CO_2_ Response in Honey Bee Hemolymph

**Figure 2:**
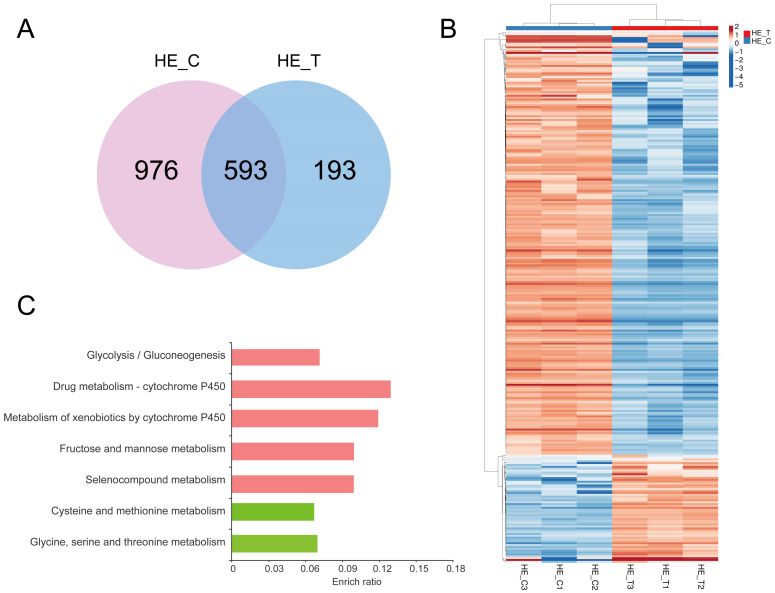
Qualitative and quantitative analysis of proteins expressed in the hemolymph of newly emerged bees. (**A**) Qualitative protein expression in the hemolymph of newly emerged bees. HE_C and HE_T represent hemolymph samples from the control and treatment groups, respectively. (**B**) Clustered heatmap of 285 quantified proteins from the hemolymph of newly emerged bees. Cluster analysis was performed using the ClustVis online tool (https://biit.cs.ut.ee/clustvis/, accessed on 10 November 2024). (**C**) Biological pathway enrichment analysis of up-regulated proteins in the hemolymph of the treatment group. Significantly enriched pathways (*p* < 0.05) were identified using the KEGG Orthology-Based Annotation System (KOBAS, http://bioinfo.org/kobas, accessed on 17 November 2024) with a hypergeometric statistical test.

**Figure 3:**
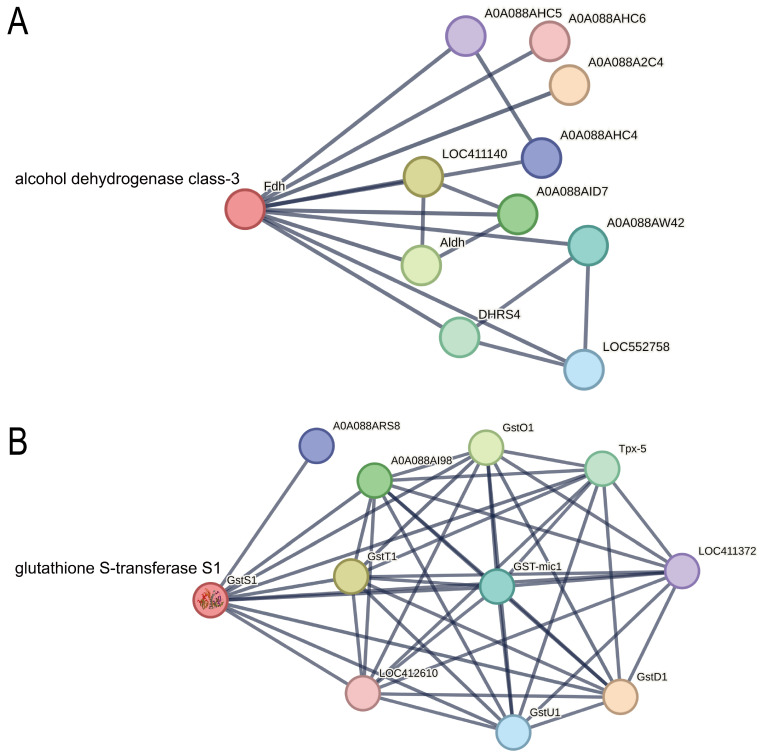
Protein–protein interaction (PPI) networks enriched in honey bee hemolymph. (**A**,**B**) Protein–protein interaction (PPI) networks for alcohol dehydrogenase class-3 (Fdh) and glutathione S-transferase S1 (GstS1) in newly emerged bees. These proteins were part of the “Drug metabolism-cytochrome P450” and “Metabolism of xenobiotics by cytochrome P450” pathways, respectively. The networks were rendered using the GeneMANIA plugin in Cytoscape (v3.9.1) to visualize interactions between upregulated proteins.

In response to CO_2_ exposure, both emerged and forager bees activated the cytochrome P450 pathway within their hemolymph, a primary route for detoxification in insects (Figure 2C and Figures S2C–S4). This pathway is vital for metabolizing both endogenous and exogenous substances [47]. The activation of this pathway was confirmed at the protein level by the elevated expression of two key enzymes: glutathione S-transferase (GST) and alcohol dehydrogenase (ADH) (Figure 3A,B). The upregulation of GST is particularly significant, as it is a critical antioxidant enzyme in bees. By contributing to the synthesis of glutathione, GST plays a direct role in neutralizing peroxides and protecting cells from the oxidative damage that can be induced by hypoxic stress [48,49].

**Figure 4:**
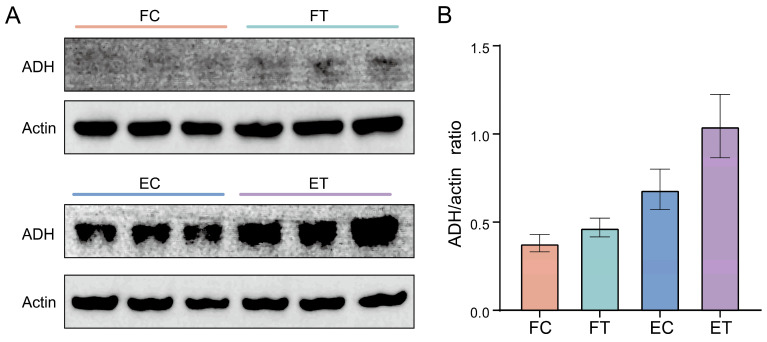
Validation of alcohol dehydrogenase (ADH) upregulation by Western Blot (WB). (**A**) WB showing the relative expression of ADH in forager (F) and newly emerged (E) bees from control (C) and treatment (T) groups. Actin was used as a loading control. (**B**) Normalized expression levels of ADH in forager and newly emerged bees. Error bars represent the standard deviation.

Similar to glutathione S-transferase, alcohol dehydrogenase (ADH) was also upregulated to cope with hypoxic stress. Specifically, ADH promotes the detoxification of exogenous methanol and protects cells from genotoxicity induced by endogenous formaldehyde, which can be produced in hypoxic environments like those created by CO_2_ exposure [50,51,52]. Furthermore, ADH works in concert with glutathione to efficiently catalyze formaldehyde, providing a sufficient antioxidative capacity that is crucial for bees to resist oxidative damage from CO_2_ stimuli [53,54,55]. These expression changes were validated by Western Blot (Figure 4). Although no significantly enriched pathways related to cytochrome P450 were found in the hemolymph of nurse bees, a different crucial protein, sorbitol dehydrogenase (SDH), was identified. The upregulation of SDH after CO_2_ exposure reflects an alternative strategy for managing oxidative stress. The primary role of SDH is to regulate the conversion of sorbitol, which helps maintain intracellular osmotic balance [56,57]. In addition, SDH plays a critical role in the oxidative stress response in nurse bees by catalyzing the production of NADH. Ultimately, these NADH molecules help bees eliminate excess free radicals, thereby protecting cells from oxidative damage [58].

### 3.3. Different Strategies Adopted by Honey Bee Brain to Respond to CO_2_ Exposure

**Figure 5:**
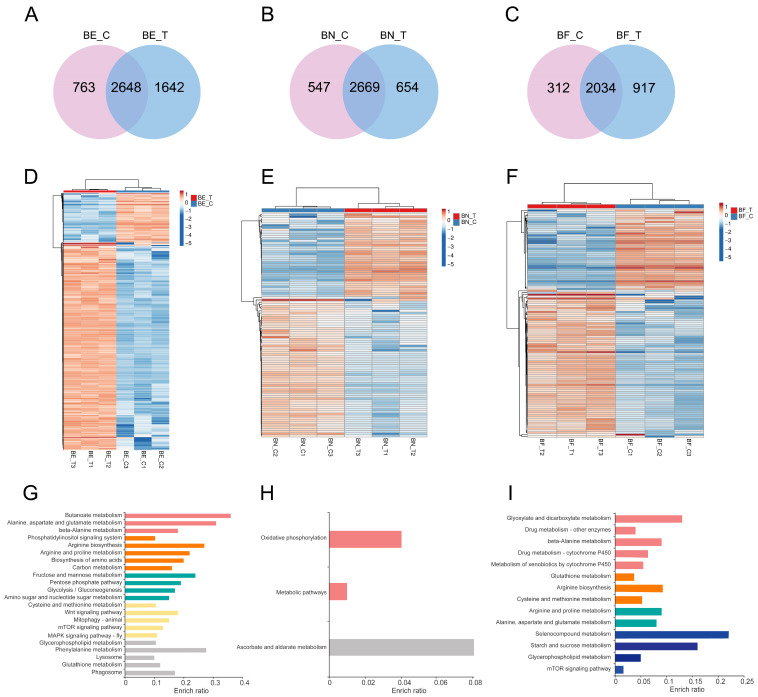
Qualitative and quantitative analysis of proteins expressed in the brains of worker bees. (**A**–**C**) Qualitative protein expression in the brains of newly emerged, nurse, and forager bees, respectively. BE, BN, and BF represent brain samples from each caste, while “C” and “T” denote control and treatment groups. (**D**–**F**) Clustered heatmaps of quantified brain proteins from newly emerged, nurse, and forager bees, respectively. (**G**–**I**) Biological pathway enrichment analysis of up-regulated proteins in the brains of newly emerged, nurse, and forager bees in the CO_2_ treatment group compared to the control group, respectively. Pathway enrichment was determined using a hypergeometric statistical test.

Honey bees exhibit significant individual variation in their response to environmental stress, which reflects their distinct physiological states and adaptive priorities. This is particularly evident in newly emerged bees, which undergo systemic reorganization and repair. Their developing brains are especially sensitive and show the most dramatic and extensive response to stress. Consequently, to protect these vulnerable and developing neural tissues from damage, newly emerged bees mount a powerful antioxidant defense centered on Glutathione metabolism (Figure 5G) [48,59].

Simultaneously, these bees initiate a large-scale “Cellular Clearance Program” involving the Phagosome, Lysosome, and Mitophagy pathways to remove damaged components (Figure 5G). More profoundly, the brain undergoes extensive signaling reprogramming. This is evidenced by the upregulation of the Wnt and mTOR signaling pathways, which are fundamental to neurodevelopment, plasticity, and cell growth [60,61,62,63,64,65]. The activation of the MAPK signaling pathway—a key mediator of stress and inflammatory responses [66,67]—further indicates a comprehensive effort to repair damage and remodel the neural network. While this adaptive remodeling is critical for survival, it may come at the cost of disrupting the bee’s normal developmental trajectory. This multi-pronged approach, combining antioxidant defense, cellular cleanup, and profound signaling reprogramming, demonstrates that the core strategy in the brain of a newly emerged bee is not merely metabolic compensation, but a fundamental reorganization to survive a crisis.

Unlike the systemic reorganization seen in newly emerged bees, nurse bees adopted a more targeted defensive strategy. They concentrated on fortifying their antioxidant capacity by upregulating pathways like ascorbate and aldarate metabolism and key components of oxidative phosphorylation (Figure 5H) [68]. This focused response was evident at the protein level, with the upregulation of several critical proteins, such as Stunted and subunits of cytochrome c oxidase and ATP synthase. By prioritizing the stability of essential physiological functions while avoiding large-scale metabolic changes, this strategy represents an efficient and precise defense mechanism tailored to minimize systemic disruption.

In response to stress, forager bees employed a strategy of precise metabolic adjustment. They broadly activated pathways related to amino acid metabolism, including those for alanine, aspartate, glutamate, cysteine, and methionine (Figure 5I). This response serves a dual purpose: it supplies precursors for key antioxidants like glutathione while also fine-tuning intermediate metabolism [69]. To further bolster their defenses, foragers also upregulated seleno-compound metabolism and reinforced membrane integrity by activating glycerophospholipid metabolism (Figure 5I) [70,71].

Collectively, this strategy centers on targeted component maintenance and metabolic fuel switching. The goal is to ensure functional resilience under sustained pressure without initiating the profound, development-related signaling reorganization observed in newly emerged bees. This represents a mature and efficient adaptation focused on immediate survival and function.

### 3.4. Intensive Energy Make Up for the Surge Requirements of Organ in CO_2_ Stress

Honeybee hemolymph and brain tissues both enhance energy metabolism to cope with CO_2_ stress, though the strategies employed differ significantly based on social caste. These responses range from adjustments in basic energy supply to the utilization of diverse substrates.

Newly emerged bees, being particularly vulnerable, adopt a strategy focused on rapid, emergency energy production. Both their hemolymph and brain tissues strongly upregulate glycolysis/gluconeogenesis and fructose/mannose metabolism, prioritizing fast ATP generation through anaerobic glycolysis (Figure 2C and Figure 5G). Concurrently, their brains activate the pentose phosphate pathway to produce NADPH, which is essential for antioxidant defense [72,73]. This activation is evidenced by the increased expression of key enzymes like phosphoglucomutase and 6-phosphogluconate dehydrogenase. To sustain this high-energy response, these bees also extensively adjust carbon metabolism and amino acid biosynthesis, replenishing metabolic intermediates to fuel a comprehensive emergency mobilization [74,75].

In contrast to other castes, the physiologically stable nurse bees demonstrated a highly precise energy management strategy. Their hemolymph showed minimal metabolic compensation, indicating a lack of a systemic, body-wide response. Instead, the brain acted specifically, upregulating the oxidative phosphorylation pathway (Figure 5H). This reflects a targeted investment to maintain efficient mitochondrial energy output, directly counteracting the inhibitory effects of CO_2_ at the most critical site.

Experienced forager bees mobilized a highly diversified energy metabolism network in both their hemolymph and brain. To expand their fuel sources beyond simple glycolysis, they significantly upregulated starch and sucrose metabolism to draw on glycogen reserves. Furthermore, they activated glyoxylate and dicarboxylate metabolism, suggesting a capacity to convert non-carbohydrate substrates, such as lipids, into energy (Figure S2C and Figure 5I). This multi-substrate strategy was further supported by the upregulation of pathways like galactose metabolism (Figure S2C).

In addition to carbohydrates and lipids, foragers also leveraged amino acids for energy. Key pathways, including alanine, aspartate, and glutamate metabolism, were upregulated in both the brain and hemolymph, involving enzymes like mitochondrial aspartate aminotransferase. This dual-purpose strategy not only provides intermediates to sustain the TCA cycle but also supplies precursors for synthesizing detoxification molecules [76,77,78].

In summary, honey bees exhibit remarkable, caste-specific strategies to manage energy metabolism under CO_2_ stress. The response of newly emerged bees is one of emergency mobilization, prioritizing basal sugar metabolism and rapid energy production to survive the initial crisis. Nurse bees adopt a more conservative and precise approach, focusing on reinforcing the efficiency of their core aerobic respiration to maintain stability. In contrast, experienced forager bees leverage their metabolic flexibility, activating a broad network to enable fuel switching and the efficient utilization of diverse energy substrates.

## 4. Conclusions

In conclusion, this work reveals that the tolerance and molecular response of adult worker honey bees to CO_2_ correlate closely with their developmental stage. New emerging bees exhibit the strongest resilience to CO_2_, which is supported by the P450 pathway in their hemolymph and strategies for antioxidation and damage repair in their brains. Nurse bees engage in low metabolic compensation in their hemolymph and adopt targeted defensive strategies in their brains. In forager bees, pathways related to detoxification in the hemolymph and metabolic regulation in the brain support their response to carbon dioxide exposure. A common thread across all stages was the strategic reallocation of energy resources to compensate for the physiological demands of CO_2_ stress. These insights not only provide a crucial theoretical basis and practical data for improving honey bee colony management and transportation protocols, but also contribute to the fundamental understanding of insect stress biology, offering a valuable model for age-dependent physiological responses in other species.

## Data Availability

All mass spectrometry proteomics datasets are publicly available in the ProteomeXchange Consortium database. The raw data and processed results have been deposited via the iProX partner repository (http://proteomecentral.proteomexchange.org, accessed on 8 September 2025) under the accession number PXD068199 [40,41].

## Supplementary Materials

The following supporting information can be downloaded at: https://www.mdpi.com/article/10.3390/insects17060630/s1, Figure S1: Qualitative and quantitative analysis of proteins expressed in the hemolymph of nurse bees; Figure S2: Qualitative and quantitative analysis of proteins expressed in the hemolymph of forager bees; Figure S3: Representative biological pathways enriched in the hemolymph of newly emerged bees following treatment; Figure S4: Representative biological pathways enriched in the hemolymph of forager bees following treatment; Table S1: Proteins Identified in the Hemolymph and Brain of Worker Bees; Table S2: Proteins Identified in the Hemolymph of Worker Bees; Table S3: Proteins Identified in the Brain of Worker Bees; Table S4: Differentially Expressed Proteins Identified (Fold change > 1.5 and *p* < 0.05) in the Hemolymph of Newly Emerged Bees.; Table S5: Biological pathway enrichment of up-regulated proteins identified in the hemolymph of ET and EC; Table S6: Differentially Expressed Proteins Identified (Fold change > 1.5 and *p* < 0.05) in the Hemolymph of Nurse Bees; Table S7: Biological pathway enrichment of up-regulated proteins identified in the hemolymph of NT and NC; Table S8: Differentially Expressed Proteins Identified (Fold change > 1.5 and *p* < 0.05) in the Hemolymph of Forager Bees; Table S9: Biological pathway enrichment of up-regulated proteins identified in the hemolymph of FT and FC; Table S10: Differentially Expressed Proteins Identified (Fold change > 1.5 and *p* < 0.05) in the Brain of Newly Emerged Bees; Table S11: Biological pathway enrichment of up-regulated proteins identified in the brain of ET and EC; Table S12: Differentially Expressed Proteins Identified (Fold change > 1.5 and *p* < 0.05) in the Brain of Nurse Bees; Table S13: Biological pathway enrichment of up-regulated proteins identified in the brain of NT and NC; Table S14: Differentially Expressed Proteins Identified (Fold change > 1.5 and *p* < 0.05) in the Brain of Forager Bees; Table S15: Biological pathway enrichment of up-regulated proteins identified in the brain of FT and FC; Original Images: original WB of EC ET_ADH; EC ET_Actin; FC FT_ADH; FC FT_Actin.
