# Supplementary material for: Effects of Carbon Dioxide on Hemolymph and Brain Proteomes in Honey Bee Workers (Apis mellifera L.)
Source: Insects. 2026 Jun 15;17(6):630. doi: 10.3390/insects17060630 (PMC13300576; doi:10.3390/insects17060630)
Supplement: Supplementary file 1 [file insects-17-00630-s001.zip › Supplemental Figures and Legends.pdf]

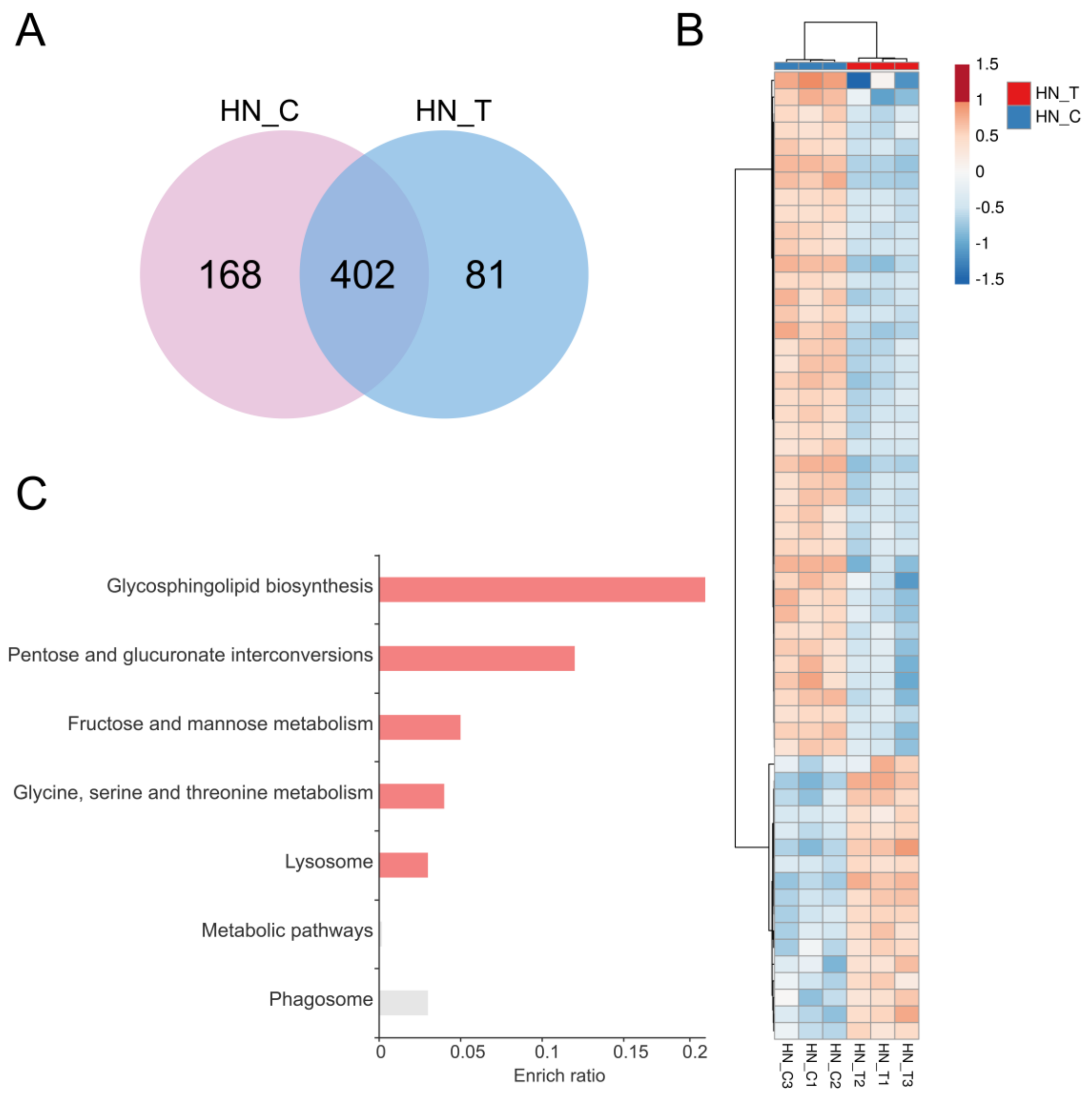

Figure S1. Qualitative and quantitative analysis of proteins expressed in the hemolymph of nurse bees. (A) Qualitative protein expression in hemolymph samples. HN represents samples from nurse bees, while "C" and "T" denote the control and treatment groups, respectively. (B) Clustered heatmap of 58 quantified proteins. Detailed results are presented in Supplemental Table S6. (C) Biological pathway enrichment analysis of up-regulated proteins in the treatment group. Pathway enrichment was determined using a hypergeometric statistical test.

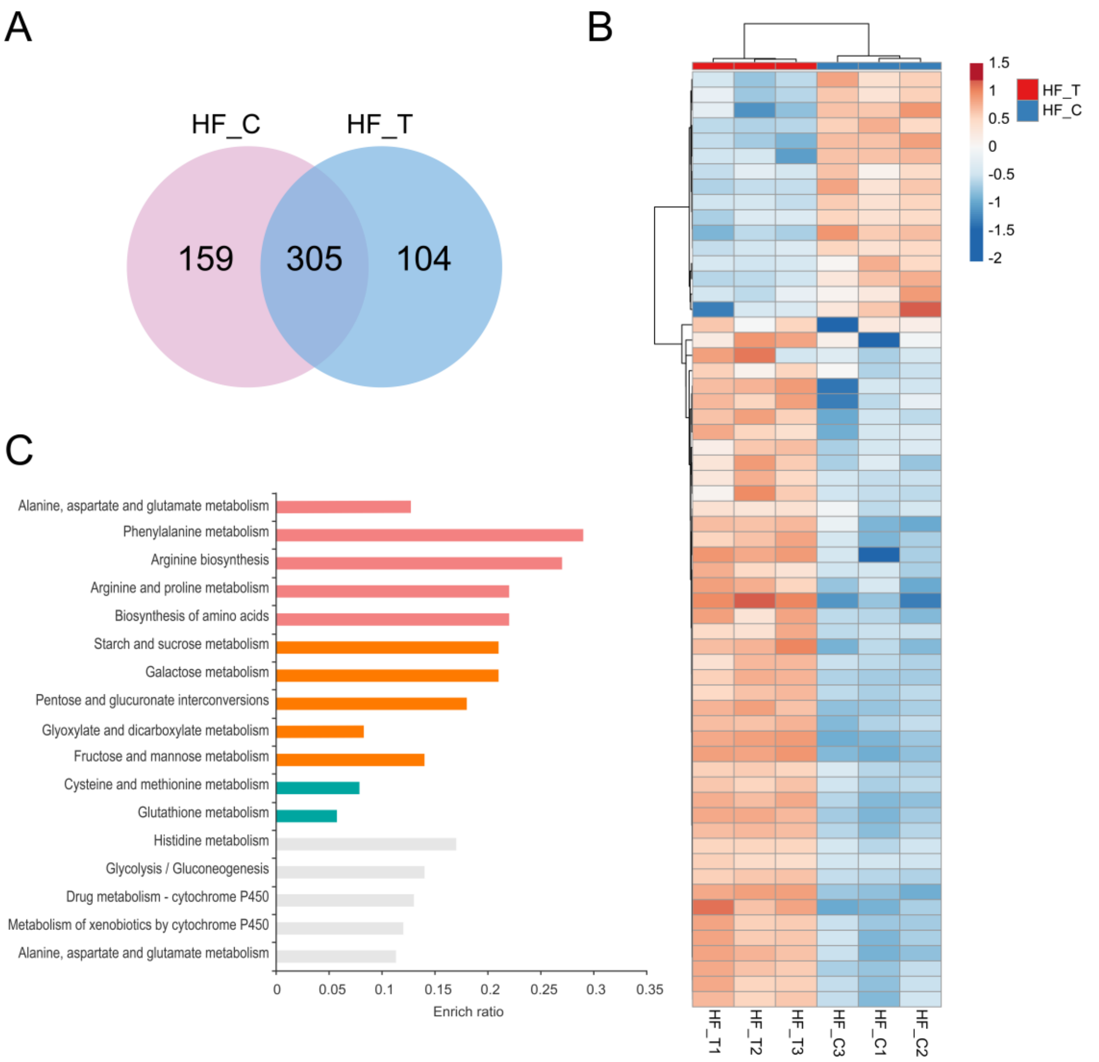

Figure S2. Qualitative and quantitative analysis of proteins expressed in the hemolymph of forager bees. (A) Qualitative overview of protein expression in hemolymph samples from forager bees (HF). "C" and "T" denote control and treatment groups. (B) Hierarchical clustering heatmap of 61 quantified proteins. See Supplemental Table S8 for detailed data. (C) KEGG pathway enrichment analysis of proteins significantly up-regulated in the treatment group (hypergeometric test, $p < 0.05$).

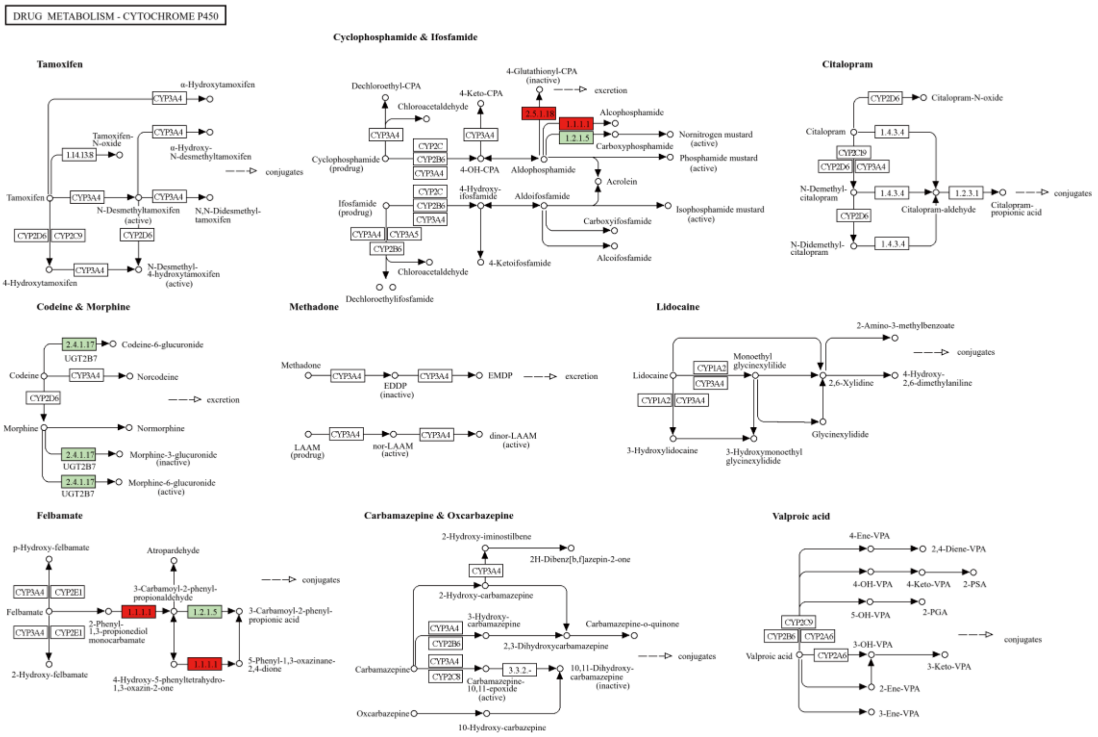

Figure S3. Representative biological pathways enriched in the hemolymph of newly emerged bees following treatment. The "Drug Metabolism Cytochrome P450" pathway was enriched by upregulated proteins. Pathway maps were sourced from KOBAS. Green-labeled boxes represent honey bee protein references annotated in the KEGG database, while red-highlighted boxes represent proteins detected in the hemolymph samples that were significantly upregulated and mapped to these enriched pathways.

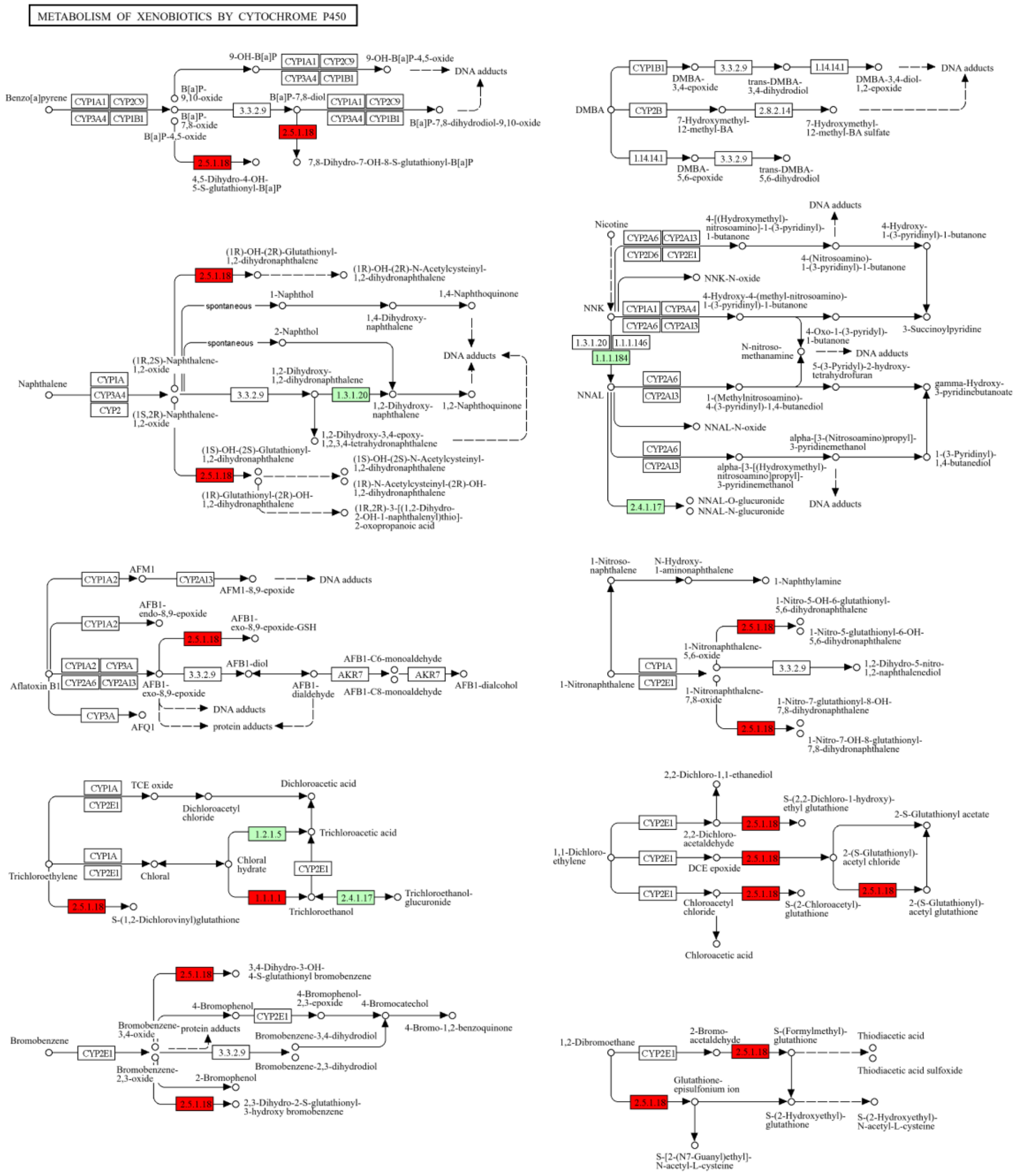

Figure S4. Representative biological pathways enriched in the hemolymph of forager bees following treatment. The "Metabolism of xenobiotics by cytochrome P450" pathway was significantly enriched by upregulated proteins. Pathway maps were sourced from KOBAS. Green-labeled boxes represent honey bee protein references annotated in the KEGG database, while red-highlighted boxes represent proteins detected in the hemolymph samples that were significantly upregulated and mapped to these enriched pathways.
